# Extended Endoscopic Endonasal Approaches for Cerebral Aneurysms: Anatomical, Virtual Reality and Morphometric Study

**DOI:** 10.1155/2014/703792

**Published:** 2014-01-19

**Authors:** Alberto Di Somma, Matteo de Notaris, Vita Stagno, Luis Serra, Joaquim Enseñat, Isam Alobid, Joan San Molina, Joan Berenguer, Paolo Cappabianca, Alberto Prats-Galino

**Affiliations:** ^1^Department of Neurosciences, Reproductive and Odontostomatological Sciences, Division of Neurosurgery, Università degli Studi di Napoli Federico II, Via Sergio Pansini 5, 80131 Naples, Italy; ^2^Laboratory of Surgical Neuroanatomy (LSNA), Faculty of Medicine, Universitat de Barcelona, Villarroel 170, 08036 Barcelona, Spain; ^3^Division of Neurosurgery, Hospital Clinic de Barcelona, Faculty of Medicine, Universitat de Barcelona, Villarroel 170, 08036 Barcelona, Spain; ^4^Center for Computational Imaging & Simulation Technologies in Biomedicine (CISTIB), Information & Communication Technologies Department, Universitat Pompeu Fabra (UPF), Tànger 122-140, 08018 Barcelona, Spain; ^5^Department of Otorhinolaryngology, Rhinology Unit, Hospital Clinic de Barcelona, Faculty of Medicine, Universitat de Barcelona, Villarroel 170, 08036 Barcelona, Spain; ^6^Medical Sciences Department, Faculty of Medicine, University of Girona, Emili Grahit 77, 17071 Girona, Spain; ^7^Department of Radiology, Neuroradiology Division, Hospital Clinic of Barcelona, Villarroel 170, 08036 Barcelona, Spain

## Abstract

*Introduction*. The purpose of the present contribution is to perform a detailed anatomic and virtual reality three-dimensional stereoscopic study in order to test the effectiveness of the extended endoscopic endonasal approaches for selected anterior and posterior circulation aneurysms. *Methods*. The study was divided in two main steps: (1) simulation step, using a dedicated Virtual Reality System (Dextroscope, Volume Interactions); (2) dissection step, in which the feasibility to reach specific vascular territory via the nose was verified in the anatomical laboratory. *Results*. Good visualization and proximal and distal vascular control of the main midline anterior and posterior circulation territory were achieved during the simulation step as well as in the dissection step (anterior communicating complex, internal carotid, ophthalmic, superior hypophyseal, posterior cerebral and posterior communicating, basilar, superior cerebellar, anterior inferior cerebellar, vertebral, and posterior inferior cerebellar arteries). *Conclusion*. The present contribution is intended as strictly anatomic study in which we highlighted some specific anterior and posterior circulation aneurysms that can be reached via the nose. For clinical applications of these approaches, some relevant complications, mainly related to the endonasal route, such as proximal and distal vascular control, major arterial bleeding, postoperative cerebrospinal fluid leak, and olfactory disturbances must be considered.

## 1. Introduction

During the last decade, the management of intracranial aneurysms has moved from neurosurgical clipping to endovascular treatment as the preferred strategy. As a matter of fact, in many world renowned neurosurgical hospitals and centers, endovascular coiling has replaced neurosurgical clipping as the treatment of choice, when coiling is technically feasible [1]. Indeed, since the publication of the International Subarachnoid Aneurysm Trial, there has been a paradigm shakeup in the management of intracranial aneurysms, and more aneurysms are referred for endovascular coiling [2]. What is apparent in the pertinent literature and discussions is that a substantial amount of controversy still exists regarding the best therapeutic strategy in patients with ruptured aneurysms. As a matter of fact, in many centers worldwide endovascular techniques majorly replaced surgery for aneurysms; however, it is not possible to lay down definitive rules for the lack of consensus studies. On the other hand, single institutions experiences have been recently published [3–6]. In a recent single-center series, it was found that 87.5% of aneurysmal subarachnoid hemorrhage patients were treated with endovascular techniques, while 12.5% with craniotomy and clip ligation, thus demonstrating the amount of shifting toward the endovascular therapy [7].

Undoubtedly, the successful development and implementation of endovascular therapy have led to a less invasive approach to cerebrovascular disease and the room for surgery has been progressively confined.

On the other side, with the rapid development of the endoscopic endonasal technique, the interest in extended transsphenoidal approaches has been renewed for many pathological entities [8–10].

Recently, few specialized centers offered to treat selected patients through a less invasive approach, such as the extended endoscopic endonasal. This new approach carries a real clinical applicability and some papers have already been published in the pertinent literature. Since the pioneering innovative works presented by Kassam et al. [11, 12], a variety of vascular lesions, mainly posterior circulation aneurysm, have started to be approached through the transsphenoidal route [13–19]. In particular, the transtuberculum-transplanum route has been used in the recent years with the aim of clipping aneurysms arising from the superior hypophyseal, anterior communicating, ophthalmic, and paraclinoidal carotid arteries [12, 17–19]. In addition, concerning posterior cerebral circulation aneurysms, other authors published cases of vertebral, basilar, and vertebral-posterior inferior cerebellar arteries aneurysms treated through endoscopic endonasal or microscopic sublabial transclival approach [11, 13–16].

Moreover, recent anatomical studies and clinical reports have detailed the extended endoscopic endonasal transsphenoidal technique, demonstrating its utility for the management of selected midline intracranial aneurysms.

Applied to vascular surgery, the endoscopic endonasal approach offers some advantages due to the properties of the endoscope itself; the endoscopic endonasal approach offers the same benefits of the endoscopic-assisted microsurgical technique used to treat cerebral aneurysms resulting in a better exposure “around the aneurysm” in order to visualize blind corners, to preserve other vessels, and to be sure of full exclusion of the aneurysm from the main cerebral circulation. Moreover, it provides a wider, close-up view of the surgical field thus allowing a close and detailed visualization of the main neurovascular structures, that is, small perforator arteries without any brain retraction.

The purpose of the present anatomic study is to verify the usefulness of the extended endoscopic endonasal approaches to access selected anterior and posterior circulation aneurysms; in other words, the aim of the present feasibility study is to determine and select cerebral circulation aneurysms that could be managed through the extended endoscopic endonasal approaches.

The vascular exposure provided by the endonasal approaches has been firstly analyzed using a preoperative 3D model with the aid of advanced virtual reality system, based on conventional computed-tomography angiography of adult patients with intracranial aneurysms arising from different cerebral vascular regions. After that, six specimens were dissected in the anatomical laboratory with the aid of a neuronavigation system, in order to verify the vascular exposure gained using the same surgical approaches.

Specifically, three main routes were identified: the transtuberculum-transplanum, the superior transclival, and the inferior transclival pathways.

## 2. Materials and Methods

### 2.1. Computed-Tomography Images Acquisition

All specimens underwent a predissection computed-tomography scan with a multislice helical acquisition protocol (slice thickness: 0,6 mm; gantry angle 0°); heads were positioned in the scanner (Siemens SOMATOM Sensation 64) in order to obtain a projection perpendicular to the palate. The images achieved were subsequently stored into a PACS (Picture Archiving and Communication System).

To allow the coregistration with the neuronavigation system, five screws were implanted in the skull as permanent bone reference markers.

### 2.2. The Dextroscope and Data Acquisition, Visualization, and Measurement

Before proceeding with the cadaveric dissection, a Virtual Reality System, known as Dextroscope (Dextroscope; Volume Interactions Pte. Ltd., Singapore), was used to perform a virtual simulation of each surgical approach. Such tool is a holographic imager that permits detailed analysis of the skull base anatomy throughout integration and fusion of multiple tomographic images series derived from the multislice computed tomography.

It provides the opportunity to perform surgical planning and decision making for different neurosurgical approaches with the aim of evaluating the best one among them. The surgeon can define and/or revise the trajectory or add a new path using interactive 3D visualization. Indeed, the virtual workspace inside the Dextroscope provides all the necessary tools for surgical planning. The hardware and software have been previously described exhaustively [20, 21].

The patient-specific radiology data sets were acquired and processed as described as follows. Each computed-tomography image set was stored in order to create a database of Digital Imaging and Communications in Medicine (DICOM) files. This database was loaded into the Dextroscope in order to build up a tridimensional model of the patient. With the aid of color-adjustment tool, it was possible to adjust individual color and transparency to distinctly reconstruct the cranial bone, soft tissue, and cerebral arteries.

For this study, three extended endoscopic endonasal approaches were planned and simulated using the Dextroscope workstation: the transtuberculum-transplanum, the superior transclival, and the inferior transclival.

The following measurements were taken in order to standardize each procedure: (a) angle of attack for each approach, defined as the angle formed by two lines of which the first beginning at the lower margin of the nostril and running parallel to the hard palate and the second originating from the same lower margin of the nostril until the selected aneurysm (its midpoint); (b) size of the craniectomy; (c) length of the surgical corridor to reach the selected aneurysm (its midpoint).

In particular, for the transtuberculum-transplanum approach, the medial edges of the optic canal were considered as lateral limits of the craniectomy, while the origin of the posterior ethmoidal artery as the upper limit and the floor of the sella as the lower one.

For the upper clivus, the paraclival carotid protuberances were accounted as lateral limits of the craniectomy, whereas the floor of the sella as the upper limit and the middle third of the clivus, at the level of the dural entry of the sixth cranial nerve (which continues anteriorly with the Dorello's canal) [22], as the lower one.

Finally, for the inferior part of the clivus, the hypoglossal canals were considered as the lateral limit at this level, while the inferior third of the clivus as the lower limit and the middle third of the clivus as the upper one.

### 2.3. Anatomical Dissection

The endoscopic endonasal transsphenoidal dissections were performed at the Laboratory of Surgical NeuroAnatomy (LSNA) of the University of Barcelona (Spain). Endoscopic procedures were carried out using a high-definition camera attached to rigid endoscopes (Karl Storz GmbH and Co, Tuttlingen, Germany), 4 mm in diameter and 18 cm in length, with 0° and 45° lenses.

Six human heads were examined. The common carotid and vertebral arteries and the jugular veins were injected with red and blue colored latex, respectively.

Predissection computed-tomography data were transferred to the laboratory navigation planning workstation and point registration was performed using bone implanted screws. A registration correlation tolerance of 2 mm was considered acceptable.

Three endoscopic endonasal approaches were performed: the transtuberculum-transplanum, the superior transclival, and the inferior transclival, taking into account the same anatomical landmarks used during virtual reality three-dimensional simulation.

The anatomical dissections were carried out with the purpose of showing all the neurovascular structures implicated in the surgical paths. In a real operating room setting there is no need to plan and perform so extensive bone removal, but since this work is intended as an anatomical paper, it was considered necessary to broadly show all the landmarks of surgical interest that could be encountered.

The present contribution should be considered as a preliminary anatomical feasibility study of the endoscopic endonasal pathway for selected cerebral aneurysms. In such preliminary step we decided to not perform any comparison with the other traditional approaches currently used to treat cerebral aneurysms (pterional, orbitozygomatic, transpetrous, retrosigmoid, or far-lateral approach) in order to first demonstrate the feasibility of the endonasal route. Further investigations are required to clearly demonstrate this matter comparing different transcranial and endonasal approaches.

## 3. Results

### 3.1. Virtual Reality Three-Dimensional Stereoscopic Results

With the help of the Dextroscope *planning platform*, the surgical strategy for minimal invasive procedures was optimized thus allowing the simulation of the extended endoscopic endonasal approaches for different anterior and posterior circulation aneurysms.

The position of the head was properly adjusted depending on the extension of the approach, using Dextroscope *moving object* tool. Three main neurosurgical approaches were simulated: (1) the transtuberculum-transplanum, (2) the superior transclival, and (3) the inferior transclival. Once the approach was chosen, the surgical route was traced out, using the *surgical pathway* tool. Subsequently, with the *drill* and the *crop* tools, virtual bone removal was carried out.

**Figure 1:**
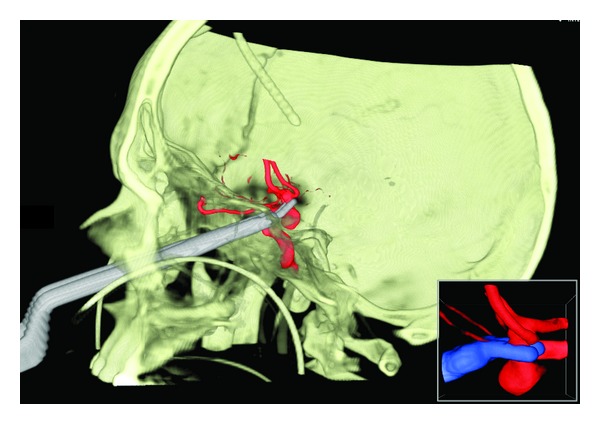
Clipping of an anterior communicating artery aneurysm via the nose in a stereoscopic virtual reality environment (patient number 1, view Table 1). Dextroscope screen shot.

As soon as the surgical route was created, a virtual clip was placed at neck of the aneurysm with the objective of achieving a virtual aneurysm clipping through an endoscopic endonasal route (Figure 1). Aneurysm clip and its specific applicator were scanned using a standard multislice CT, in order to insert them into the Dextroscope.

**Figure 2:**
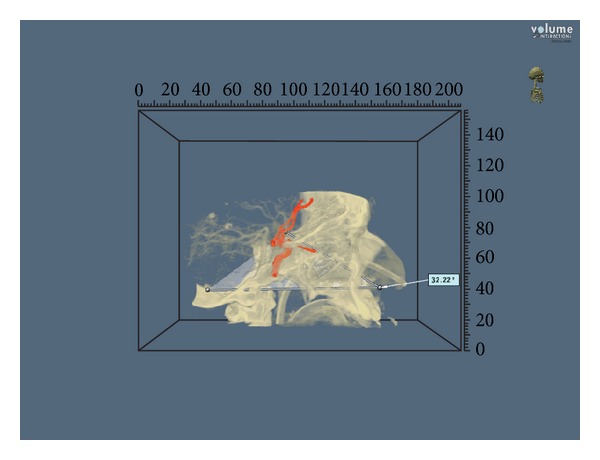
Angle of attack to an anterior communicating artery aneurysm approached via the transtuberculum-transplanum route (patient number 1, view Table 2). Dextroscope screen shot. For investigational use only.

**Figure 3:**
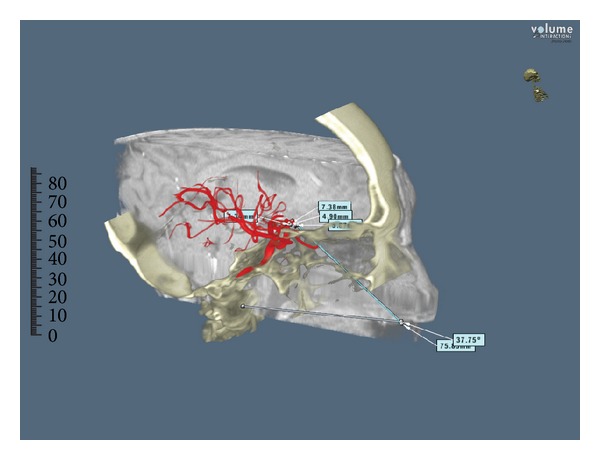
Angle of attack and length of the surgical corridor in a carotid-ophthalmic aneurysm approached via the transtuberculum-transplanum route (patient number 2, view Table 2). Dextroscope screen shot. For investigational use only.

The angle of attack, the size of the craniectomy, and the length of the surgical corridor were calculated for each approach on 4 illustrative patients with intracranial aneurysms arising from different cerebral vascular regions (Table 1) (Figures 2 and 3).

The sizes of craniectomy for the anterior communicating artery complex and the basilar tip were 7,85 cm^2^ and 3,52 cm^2^ respectively, while for the carotid-ophthalmic and VA-PICA junctions were 7,86 cm^2^ and 4,62 cm^2^, respectively.

Angles of attack to the anterior communicating artery complex and basilar tip were 32,22 and 31,4 degrees, respectively, while to the carotid-ophthalmic and VA-PICA junctions were 37,75 and 27,47 degrees, respectively. The lengths of the surgical corridor to reach the anterior communicating artery complex and the basilar tip were 87,2 mm and 92,2 mm, respectively, while for the carotid-ophthalmic and VA-PICA junctions were 75,3 mm and 89,1 mm, respectively. Results are summarized in Table 2.

### 3.2. Anatomical Results

#### 3.2.1. Transtuberculum-Transplanum Approach

The details of the transtuberculum-transplanum approach have been well cleared up elsewhere [23–25].

**Figure 4:**
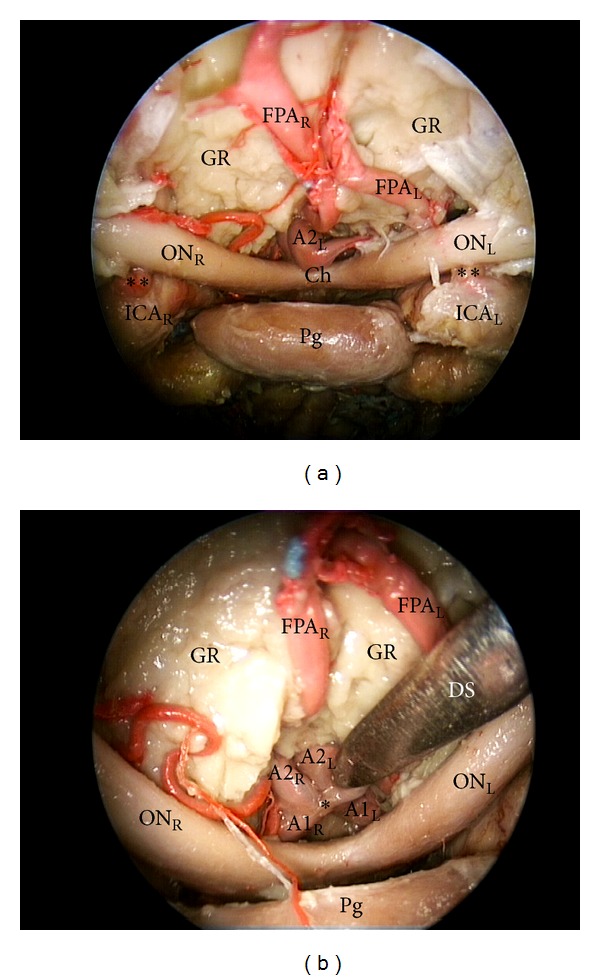
(a) Endoscopic endonasal transtuberculum-transplanum approach to the anterior brain circulation; (b) close-up view of the suprasellar area (30 degree endoscope). FPA: frontopolar artery; A1: precommunicating tract of anterior cerebral artery; A2: post communicating tract of anterior cerebral artery; ICA: internal carotid artery; ON: optic nerve; Ch: chiasm; Pg: pituitary gland; Pg: pituitary gland; GR: gyri recta; DS: dissector; L: left; R: right; ∗: anterior communicating artery; ∗∗: ophthalmic artery.

The transtuberculum-transplanum approach permitted to show the precommunicating and postcommunicating segments of the anterior cerebral arteries (A1 and A2), the anterior communicating artery (AcomA), the frontopolar arteries (FPA), the superior hypophyseal arteries (sha), the proximal segment of the ophthalmic arteries (OphA), and the supraclinoid portion of the internal carotid arteries (ICA) (Figure 4).

The exposure of a large portion of the anterior cerebral circulation anatomy, provided by this approach, allows a safe proximal and distal vascular control, for example, in case of anterior communicating artery aneurysms. Therefore, according to the basic principles enunciated by Yaşargil [26], if a temporary clip is required, it should be applied first to the larger A1 segment and placed medial to the perforating arteries.

#### 3.2.2. Superior Transclival Approach

The extended endoscopic endonasal transsphenoidal approach to the upper part of the clivus has been already described in the current literature [27].

**Figure 5:**
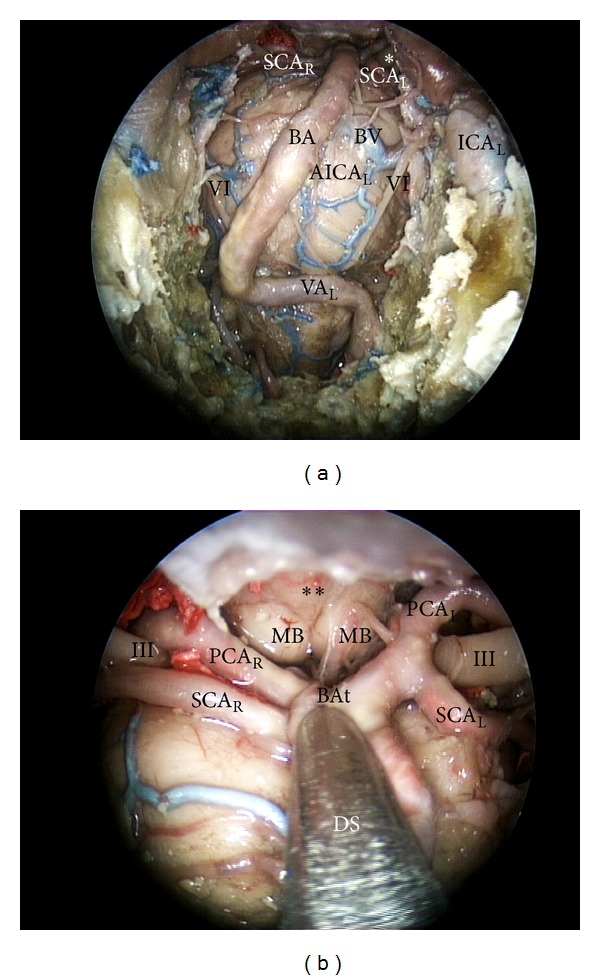
(a) endoscopic endonasal approach to the superior part of the clivus; (b) close up view of the superior third of the retroclival area. III: third cranial nerve; SCA: superior cerebellar artery; BA: basilar artery; BV: basilar vein; AICA: anterior inferior cerebellar artery; PCA: posterior cerebral artery; MB: mammilary body; BAt: tip of the basilar artery; ICA: internal carotid artery; DS: dissector; L: left; R: right; ∗: third cranial nerve; ∗∗: floor of the third ventricle.

The superior transclival approach permitted to expose the tip and the superior part of the trunk of the basilar artery (BA), the posterior cerebral arteries (PCA), the posterior communicating arteries (PcomA), the superior cerebellar arteries (SCA), the anterior inferior cerebellar arteries (AICA), and, laterally, the paraclival portion of the ICA (Figure 5).

From this ventral point of view, the exposure of the upper part of the posterior cerebral circulation enables a secure vascular control both proximal and distal, for example, in case of basilar tip aneurysms. Therefore, as illustrated by Yaşargil, if a temporary clip is required, it should be placed distal to the SCA although the SCA-PCA distance is often too short to accomplish this. Alternatively, If the bleeding is not well governed, temporal vascular control should be achieved by clipping the basilar trunk or one or both the PCA or the PcomA [26].

#### 3.2.3. Inferior Transclival Approach

The extended endonasal approach to the lower part of the clivus has been also described in the pertinent literature [28, 29].

**Figure 6:**
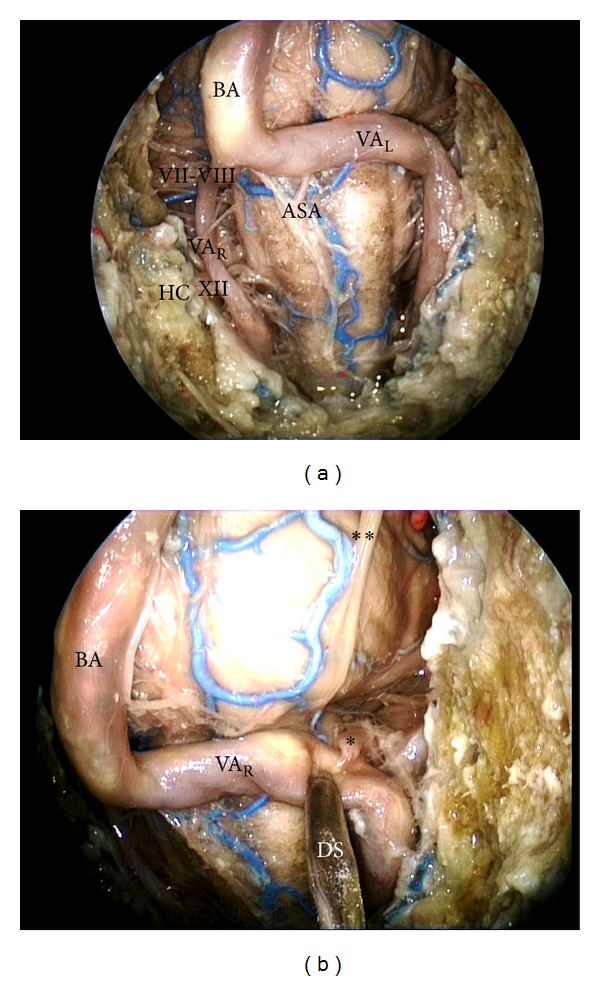
(a) Endoscopic endonasal approach to the inferior part of the clivus; (b) close-up view of the vertebral artery-posterior inferior cerebellar artery complex. BA: basilar artery; VA: vertebral artery; ASA: anterior spinal artery; VI: abducent nerve; VII-VIII: acoustic-facial nerve bundle; HC: hypoglossal canal; XII: hypoglossal nerve; DS: dissector; L: left; R: right; ∗: posterior inferior cerebellar artery; ∗∗: abducent nerve.

The transclival approach permitted to show the trunk of the basilar artery (BA), the anterior inferior cerebellar arteries (AICA), the vertebral arteries (VA), the posterior inferior cerebellar arteries (PICA), and the anterior spinal artery (ASA) (Figure 6).

The exposure of the lower part of the posterior cerebral circulation enables a direct proximal and distal vascular control, for example, in case of midline aneurysms arising from the VA-PICA junction [16]. Therefore, if a temporary clip is required in those cases, it should be placed on the basilar trunk proximally and on both vertebral artery and lateral medullary or anterior medullary segments of the posterior inferior cerebellar artery distally.

## 4. Discussion

The management of cerebral aneurysms has undergone significant evolution in the recent years. Nowadays, it can be considered as the result of a close cooperation between different specialists, giving their own contribution to the final result, specifically devoted to single patients.

During the last decades, the endovascular technique has rapidly evolved, and actually, endovascular therapy has largely replaced microsurgery as the first line treatment modality for the majority of cerebral aneurysms [2]. On the other side, regarding the surgical approach to cerebral aneurysms, it has to be said that vascular neurosurgery improved dramatically when the operating microscope was introduced in the 1960s and the pterional approach was described by Yaşargil in the 1980s [26, 30].

In spite of these great advances made both in surgical and endovascular techniques, the complexity of some vascular lesions makes their treatment still a challenge for vascular teams, as such pathologies are associated with a high incidence of complications, which is particularly true for posterior circulation aneurysms. For such reasons, starting from the late sixties, “the ventral route,” namely, the transcervical-transclival, transoral-transclival, transfacial-transclival, and, finally, extended endoscopic endonasal approaches, has been advocated as a valid alternative to reach a variety of anterior and posterior circulation aneurysms. Indeed, the ventral pathway was considered a reasonable option allowing direct access to the surgical field obviating brain retraction and, in selected cases, obtaining an early and safe proximal and distal vascular control [31].

Concerning the transsphenoidal route, Kassam et al. published the first case of cerebral aneurysm clipping via the nose [11]. Afterwards, other reports appeared in the literature stating that some specific midline anterior and posterior circulation aneurysms, not amenable to endovascular treatment, could be managed via the anterior route, in particular throughout the extended endoscopic endonasal pathway [11–19].

Nevertheless, the endoscopic endonasal surgery applied to vascular lesions brings also some disadvantages that should be taken into consideration. The exposure provided by the approach is limited to certain anatomical skull base regions, and proximal and distal vascular control may be hard in case of difficulties, but it is possible when performing the transtuberculum-transplanum, the superior transclival, or the inferior transclival approach depending on the aneurysm location on the vessel (see results paragraph). On the other hand, it should be highlighted that, in some cases, also transcranial approaches do not ensure a safe and valid proximal and distal vascular control, but it happened not so often.

Furthermore, the inability to perform a bypass graft and the significant endoscopic skills required can be considered as other important drawbacks, making the ventral corridor indicated only in very selected cases. Moreover, in the endonasal approach the surgical corridor may be narrow, with less leaving room for the surgeon to comfortably dissect and definitively clip the aneurysm. Another major issue from the extended endoscopic endonasal route still remains achieving effective cranial base repair. The widespread use of extended approaches for the removal of different skull base lesions has led to the reemergence of the postoperative CSF leak as a major complication for this kind of surgery. However, neurosurgical familiarity and experience in transsphenoidal surgery and otorhinolaryngologist expertise in the use of autologous mucosal flaps have contributed to the reduction in the incidence of postoperative CSF leaks, also after extended endoscopic endonasal approaches [10, 32].

In the present anatomical study we measured the exposition of selected anterior and posterior brain circulation aneurysms provided by three different endoscopic endonasal routes, that is, the transtuberculum-transplanum, the superior transclival, and the inferior transclival pathways.

Furthermore, we analyzed the utility of a Virtual Reality System, as the Dextroscope, to perform a virtual simulation of each surgical approach. Using the Dextroscope we gave detailed measurements for each surgical approach used in this study, calculating the angle of attack, the size of the craniectomy, and the length of the surgical corridor.

The results shown in the present study demonstrate that an extended endoscopic endonasal approach to the suprasellar area, such as the transtuberculum-transplanum route, provided a wider angle of attack to reach the carotid ophthalmic junction and the anterior communicating artery complex compared to the approaches used to reach the clivus for posterior circulation aneurysms (37,75° and 32,22° versus 31,4° and 27,47°).

Moreover, the length of the transtuberculum-transplanum corridor to get to carotid ophthalmic and anterior communicating artery aneurysms was found to be shorter compared to the other posterior circulation aneurysms cases analyzed (75,3 mm and 87,2 mm versus 92,2 mm and 89,1 mm).

Regarding carotid-ophthalmic aneurysms, it should be said that if on one hand endovascular treatment is safe and efficacious, even if associated with risks of retinal artery occlusion or delayed optic ischemia [33, 34], on the other hand the microsurgical management of these lesions can be challenging due to their proximal location and their close relationships with the cavernous sinus, carotid siphon, and optic nerve, which often cover the neck of the aneurysm, thus making clipping particularly hazardous [35–37].

Regarding vascular lesion of the anterior communicating artery complex, it should be stressed that this region has got a peculiar anatomy, including anatomic variations, multiple perforators, and a deep location which make access to aneurysms arising from those arteries considerably difficult, with respect to both microsurgical and endovascular treatments [38].

Taking into account these considerations, the endoscopic endonasal transtuberculum-transplanum approach can be evaluated as an effective and available alternative in order to treat carotid-ophthalmic and anterior communicating artery aneurysms, alone or in combination with the other different microneurosurgical and endovascular procedures.

With regard to posterior cerebral circulation aneurysms, it should be said that despite the fact that angles of attack and length of the surgical corridor obtained with endoscopic endonasal approaches to clivus were less favorable compared to the suprasellar approach for anterior circulation aneurysms, in experienced hands the endoscopic endonasal superior and inferior transclival pathways could be alternative for selective and more accessible basilar tip and VA-PICA complex aneurysms, not amenable to endovascular treatment [13–16]. It must be remarked that the endovascular approach has to be considered as the first line of treatment, whereas the endonasal route could be an alternative, a second or even a third line strategy, for small posterior cerebral circulation aneurysms, with ventral orientation and midline position.

Indeed, endoscopic endonasal approaches to the clivus allow direct exposure of the posterior cerebral circulation system via minimal craniectomies compared to the other anterior circulation aneurysms cases shown in this study (3,52 cm^2^ and 4,62 cm^2^ versus 7,85 cm^2^ and 7,86 cm^2^), thus avoiding more extensive skull base approaches which could require complex and larger craniotomies, demanding a certain neurovascular manipulation to gain an adequate intradural exposure, even though the lesion has small dimensions and is located in the midline.

In the present study we determined that the most feasible anterior circulation aneurysms that could be managed through the extended endoscopic endonasal approaches are selected anterior cerebral, communicating, ophthalmic, and superior hypophyseal arteries aneurysms. Concerning posterior circulation, the basilar, proximal superior cerebellar, anterior inferior cerebellar, posterior inferior cerebellar, and vertebrobasilar junction artery segments can be exposed as well. This paper is intended as strictly anatomic study in which we highlighted some specific anterior and posterior circulation aneurysms that can be reached via the nose.

For clinical applications of these approaches, some relevant complications, mainly related to the endonasal route, such as bleeding control, postoperative cerebrospinal fluid leak, and olfactory disorders must be considered. In the future, the endonasal routes could be taken into account as a routinely valid alternative for selected anterior and posterior circulation aneurysms, not amenable to endovascular and/or microneurosurgical treatment.

## 5. Conclusions

Advances in endoscopic skull base surgery have increased our ability to access to complex cranial base and, more recently, vascular lesions. However, there is no doubt that aneurysm surgery is greatly advanced with the advent of endovascular techniques and the reserved space for open surgery has been and will be progressively limited.

Anyway, in specialized centers, where extended transsphenoidal approaches are routinely performed, selected cases may be treated with such techniques alone or in combination with other different procedures.

## Figures and Tables

**Table 1:** List of 4 patients with different intracranial aneurysms selected for the virtual reality three-dimensional stereoscopic study.

Patient	Aneurysm location	Direction	Form	LL (mm)	AP (mm)
1	AcomA	Downward	Saccular	7.85	7.46
2	Carotid-ophthalmic junction	Anterior, medial, downward	Saccular	7.36	5.38
3	Basilar tip	Upward	Saccular	5.42	6.83
4	VA-PICA junction	Upward, anterior	Saccular	1.2	1.2

AcomA: anterior communicating artery; VA-PICA: vertebral artery-posterior inferior cerebellar artery; LL: laterolateral diameter of the aneurysm; AP: anteroposterior diameter of the aneurysm.

**Table 2:** List of the 4 approaches used to reach different intracranial aneurysms, and measurement taken in order to standardize each procedure.

Patient	Approach	Aneurysm	Craniectomy (cm^2^)	AOA (°)	LSC (mm)
1	Transtuberculum-transplanum	AcomA	7.85	32.22	87.2
2	Transtuberculum-transplanum	Carotid-ophthalmic junction	7.86	37.75	75.3
3	Superior transclival	Basilar tip	3.52	31.4	92.2
4	Inferior transclival	VA-PICA junction	4.62	27.47	89.1

AcomA: anterior communicating artery; VA-PICA: vertebral artery-posterior inferior cerebellar artery; AOA: angle of attack; LSC: length of the surgical corridor.
